# Cannabinoid Ligands Targeting TRP Channels

**DOI:** 10.3389/fnmol.2018.00487

**Published:** 2019-01-15

**Authors:** Chanté Muller, Paula Morales, Patricia H. Reggio

**Affiliations:** Department of Chemistry and Biochemistry, University of North Carolina at Greensboro, Greensboro, NC, United States

**Keywords:** cannabinoids, TRP channels, cannabidiol, TRPV1, TRPA1, TRPM8

## Abstract

Transient receptor potential (TRP) channels are a group of membrane proteins involved in the transduction of a plethora of chemical and physical stimuli. These channels modulate ion entry, mediating a variety of neural signaling processes implicated in the sensation of temperature, pressure, and pH, as well as smell, taste, vision, and pain perception. Many diseases involve TRP channel dysfunction, including neuropathic pain, inflammation, and respiratory disorders. In the pursuit of new treatments for these disorders, it was discovered that cannabinoids can modulate a certain subset of TRP channels. The TRP vanilloid (TRPV), TRP ankyrin (TRPA), and TRP melastatin (TRPM) subfamilies were all found to contain channels that can be modulated by several endogenous, phytogenic, and synthetic cannabinoids. To date, six TRP channels from the three subfamilies mentioned above have been reported to mediate cannabinoid activity: TRPV1, TRPV2, TRPV3, TRPV4, TRPA1, and TRPM8. The increasing data regarding cannabinoid interactions with these receptors has prompted some researchers to consider these TRP channels to be “ionotropic cannabinoid receptors.” Although CB1 and CB2 are considered to be the canonical cannabinoid receptors, there is significant overlap between cannabinoids and ligands of TRP receptors. The first endogenous agonist of TRPV1 to be discovered was the endocannabinoid, anandamide (AEA). Similarly, *N-*arachidonyl dopamine (NADA) and AEA were the first endogenous TRPM8 antagonists discovered. Additionally, Δ^9^-tetrahydrocannabinol (Δ^9^-THC), the most abundant psychotropic compound in cannabis, acts most potently at TRPV2, moderately modulates TRPV3, TRPV4, TRPA1, and TRPM8, though Δ^9^-THC is not reported to modulate TRPV1. Moreover, TRP receptors may modulate effects of synthetic cannabinoids used in research. One common research tool is WIN55,212-2, a CB1 agonist that also exerts analgesic effects by desensitizing TRPA1 and TRPV1. In this review article, we aim to provide an overview and classification of the cannabinoid ligands that have been reported to modulate TRP channels and their therapeutic potential.

## Introduction

Transient receptor potential (TRP) channels are a superfamily of trans-membrane ion channels involved in transduction in response to a plethora of chemical and physical stimuli. Comprised of four subunits with 6 trans-membrane helices (S1–S6) each, TRP channels can homo- or heterotetramerize to create a pore for cation permeation that is located between helices 5 and 6 (Caterina, 2014). These channels are found in the plasma membrane and can gate several types of mono- and divalent cations, in single-file fashion, through the pore following exposure to a stimulus. TRP channels have also been implicated as sensors of many physiological and pathological processes including itch, temperature sensation, cancers, genetic disorders, and pain (Perálvarez-Marín et al., 2012; Vay et al., 2012; Caterina, 2014).

*Cannabis Sativa* has been used for centuries to treat ailments including chronic pain, and extensive literature precedent supports the role of phytogenic and endogenous cannabinoids as pain modulators (Caterina, 2014). Chronic pain is a significant and complex problem that encompasses many different conditions, symptoms, and pathways. Once nociceptors are stimulated, action potentials are generated and then propagated to the brain, resulting in a sensation of pain (Vay et al., 2012). Currently, the most efficient way to treat chronic pain is with opioids, however the opioid system also influences the reward center and long-term opioid usage can lead to addictive behavior (Storozhuk and Zholos, 2018). Since the etiologies related to pain and the mechanisms of action underlying hypersensitivity are diverse, targeting the ion channels that contribute to the detection of stimuli may be an effective approach in treating pain syndromes (Levine and Alessandri-Haber, 2007). Since the cloning of TRPV1, at least five other TRP channels have been discovered in the dorsal root ganglia (DRG), that can also be found in primary somatosensory neurons. These channels have been identified as sensory transducers that may participate in the generation of painful sensations evoked by thermal, mechanical, or chemical stimuli making them a desirable target in the development of treatments for chronic pain syndromes (Levine and Alessandri-Haber, 2007). One feature sought for exploitation from these TRP channels, especially TRPV1, is desensitization. TRPV1 becomes rapidly desensitized upon activation, rendering the channel refractory to further stimulation. This mechanism is thought to underlie the paradoxical analgesic effect of TRPV1 and may explain the reduced neuronal activity upon activation of other TRP channels (Iannotti et al., 2014). This paradoxical analgesic effect is the basis of capsaicin-based creams for chronic pain (De Petrocellis et al., 2011a). However, the pungency of compounds like capsaicin can cause vascular and respiratory side effects when administered systemically (Luongo et al., 2012). For this reason, the use of non-pungent compounds to activate and therefore desensitize TRP channels is desired.

Targeting the endocannabinoid system has been shown to be a promising strategy for the modulation of pain (Woodhams et al., 2017). In fact, activation of the cannabinoid receptors CB1 and CB2, as well as inhibition of endocannabinoid deactivation (blockade of endocannabinoid uptake or degradation) has shown antinociceptive responses (Guindon and Hohmann, 2009). Pharmacological evidence suggests that cannabinoids and endocannabinoids target more than the canonical cannabinoid receptors (Morales and Reggio, 2017; Morales et al., 2017, 2018). There is evidence suggesting that some TRP channels (TRPV1–4, TRPA1, and TRPM8) can be modulated by cannabinoids, providing a promising multitarget approach for the treatment of pain. Interestingly, CB1 has been suggested to colocalize with TRP channels such as TRPV1 in sensory and brain neurons (Ahluwalia et al., 2003; Price et al., 2004; Cristino et al., 2006), while CB2 colocalizes with this channel in sensory neurons and osteoclasts (Anand et al., 2008; Rossi et al., 2009). This expression pattern makes concerted actions possible to modulate nociceptive responses, as well as a synergistic functional effect of cannabinoid ligands.

The mammalian TRP superfamily consists of six subfamilies: canonical (TRPC), vanilloid (TRPV), polycystin (TRPP), mucolipin (TRPML), ankyrin (TRPA), and melastatin (TRPM; Winter et al., 2013). There are 28 channels in the TRP superfamily. Six of these channels can be activated by a variety of endogenous, phytogenic, and synthetic cannabinoids, as well as, other physical and chemical stimuli. These six channels, TRPV1-TRPV4, TRPA1, and TRPM8, are termed the *ionotropic cannabinoid receptors* and are the focus of this review.

**Figure 1:**
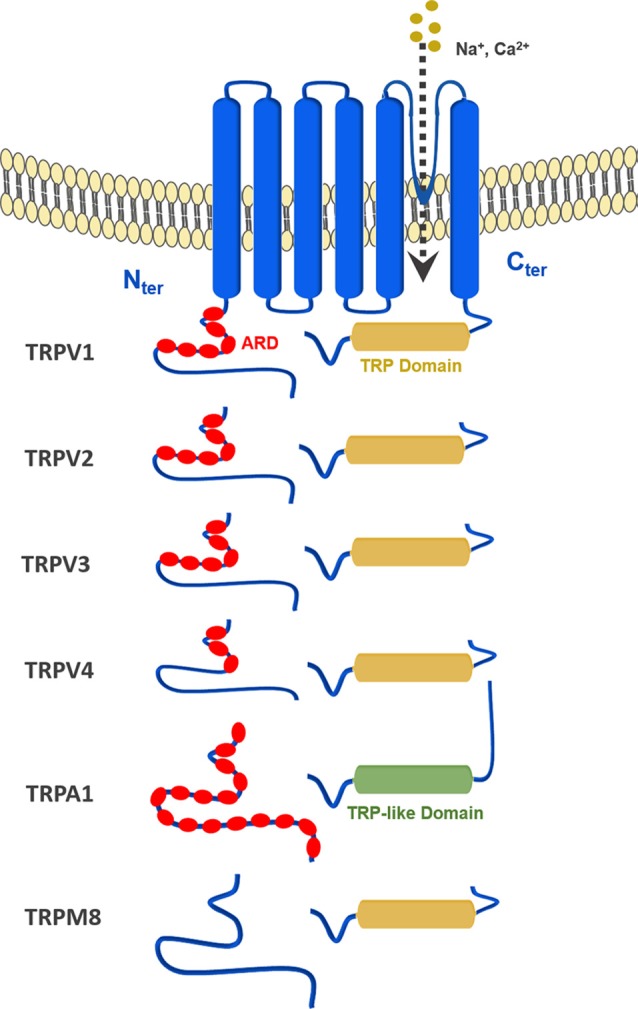
General topology of the transient receptor potential (TRP) channels discussed in this review: TRPV1–4, TRPA1 and TRPM8.

**Figure 2:**
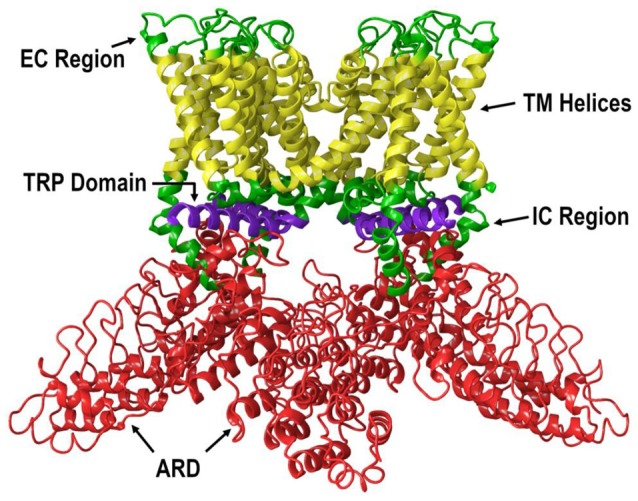
The lipid view of TRPV1 adapted from PDB: 3J5P. Ankyrin repeat domain (ARD) shown in red, transmembrane helices shown in yellow, TRP domain shown in purple, and intracellular regions (ICRs) and extracellular regions (ECRs) shown in green. Sections have been omitted for clarity.

**Figure 3:**
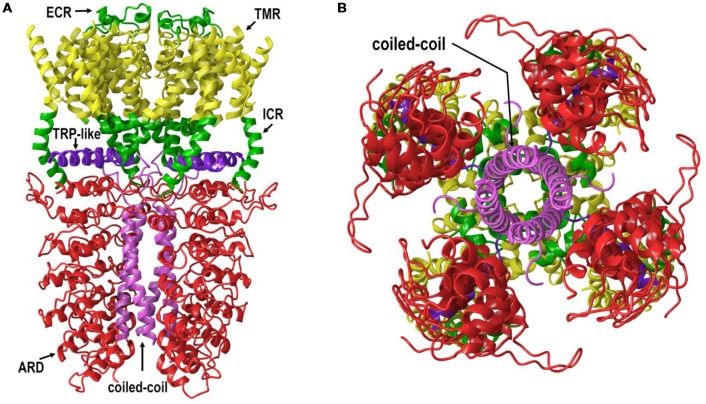
**(A)** The lipid view of TRPA1 adapted from PDB: 3J9P. ARD shown in red, transmembrane region (TMR) shown in yellow, TRP-like domain shown in purple, ICRs- and ECRs shown in green and coiled-coil shown in pink. Sections have been omitted for clarity. **(B)** The intracellular view of TRPA1 adapted from PDB: 3J9P. Coiled-coil shown in pink. Sections have been omitted for clarity.

All TRP channels have a similar topological profile: six transmembrane helices, a short pore helix, and a pore loop. However, there are some structural divergences that characterize each class of TRP channels. The main difference among the three subfamilies discussed here is the variability in the number of ankyrin repeat domains (ARDs) located at the N-terminus of the receptor. Vanilloid-type channels bear a variable number of ankyrin repeats; the Ankyrin subfamily presents a high number of repeats; and, the TRPM subfamily lacks ankyrin repeats. The topology of the channels reviewed here is depicted in Figure 1. For instance, on the N-terminal side of TRPV1 lies a series of ankyrin repeat units that form the ARD (Figure 2). Each unit contains two short anti-parallel alpha helices and a finger loop that extends out at a 90° angle from the axis of the helices (Hellmich and Gaudet, 2014). TRPV1 specifically contains six of these repeat units on each monomer that forms a concave surface used for interactions with other proteins like calmodulin (CaM) and phosphatidylinositol-3-kinase (PI3K; Nilius and Szallasi, 2014). Similarly, TRPA1 also contains an ARD and this class of ion channels was named for the unusually large number of ankyrin repeats it contains (Figure 3). One motif found in TRPA1 and TRPM8 that is not present in the vanilloid subfamily is a C-terminal tetrameric coiled-coil (Figures 3A,B) which mediates interactions between subunits and is important for trafficking and function (Paulsen et al., 2015; Yin et al., 2018). Another large structural difference between the TRPV, TRPA, and TRPM subfamilies is the TRP box. The TRP box is a long helix that is parallel to the membrane, on the C-terminal side of the receptor, and can be found in both TRPV1 and TRPM8 (Figure 2). Though not canonically present in TRPA1 due to its location farther below the inner leaflet (Figure 3A), the α-helix that extends off of the C-terminal side of the receptor is topologically and structurally analogous to a TRP box (Hellmich and Gaudet, 2014; Paulsen et al., 2015). Despite the topological differences among TRPV1-V4, TRPA1, and TRPM8, all respond to select cannabinoids and are therefore classified as ionotropic cannabinoid receptors.

**Figure 4:**
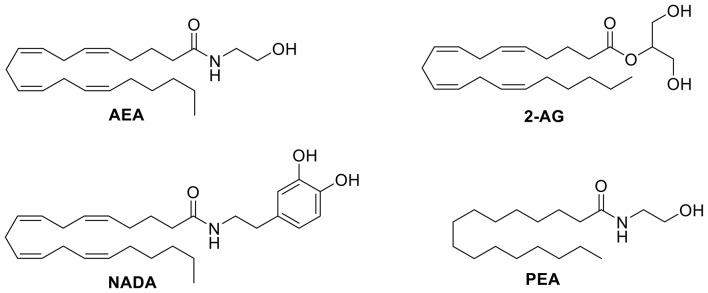
Structure of selected endocannabinoids that target TRP channels.

**Figure 5:**
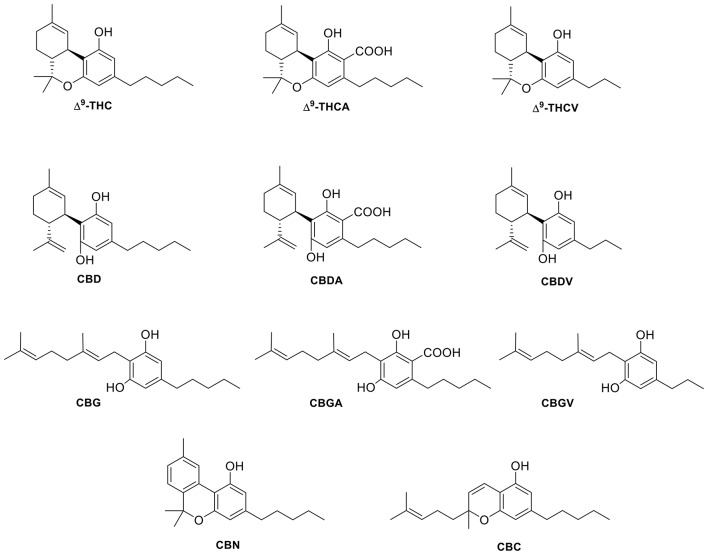
Structure of selected plant cannabinoid ligands that target TRP channels.

**Figure 6:**
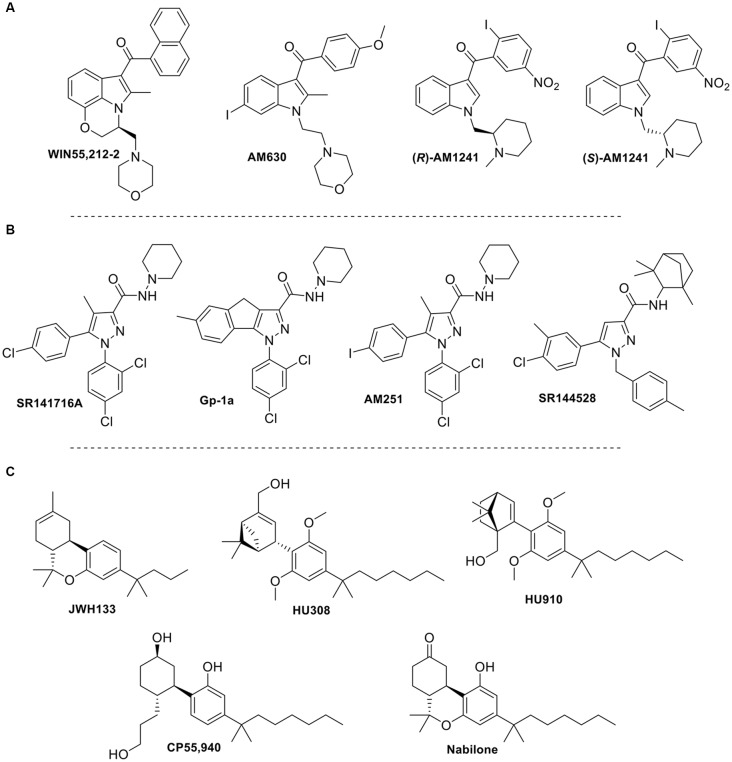
Structure of selected synthetic cannabinoid ligands that target TRP channels: **(A)** aminoalkyindole derivatives; **(B)** arylpyrazole derivatives; **(C)** synthetic phytocannabinoids analogs.

Many endogenous and exogeneous compounds activate receptors found in the TRP superfamily. Natural, pungent compounds like capsaicin and allicin, from chili peppers and garlic respectively, can activate and gate specific TRP channels. In addition to these pungent compounds, the six TRP channels that make up the ionotropic cannabinoid receptors can also be modulated by endogenous, phytogenic, and synthetic cannabinoids. For example, the endocannabinoid anandamide (AEA, Figure 4) was the first endogenous TRPV1 agonist identified during a study of the vasodilator action of AEA (Zygmunt et al., 1999). *N*-arachidonyl dopamine (NADA, Figure 4) and AEA were identified as the first endogenous antagonists of TRPM8 (De Petrocellis et al., 2007). Δ^9^-tetrahydrocannabinol (Δ^9^-THC, Figure 5) acts most potently at TRPV2; moderately modulates TRPV3, TRPV4, TRPA1, and TRPM8; but, does not appear to modulate TRPV1 (De Petrocellis et al., 2011b). Cannabidiol (CBD, Figure 5) has been shown to have many beneficial properties, including anti-inflammatory action. CBD has little affinity for the CB1 and CB2 receptors, but is reported to be most potent at TRPV1 and TRPM8 channels (De Petrocellis et al., 2011b). A common synthetic cannabinoid known for its use as a CB1 agonist, WIN55,212-2 (Figure 6), has been found to exert analgesic effects by desensitizing both TRPV1 and TRPA1 (Ruparel et al., 2011).

There are many more cannabinoid ligands that target the ionotropic cannabinoid receptors. In this review, we provide an overview and classification of the various cannabinoid ligands that modulate the ionotropic cannabinoid receptors and we explore the therapeutic potential of these ligands.

## TRPV1

TRPV1, also known as the capsaicin receptor, is a polymodal, nonselective cation channel expressed by all major classes of nociceptive neurons and is important for the detection of noxious stimuli (Vay et al., 2012; Caterina, 2014). Ion channels, including TRPV1, are typically found in the plasma membrane and form a passageway from one side of the membrane to the other (De Petrocellis et al., 2017). Upon activation, the pore of TRPV1 opens and allows ions to pass from one side of the membrane to the other. TRPV1 can be activated by a number of endogenous and exogenous stimuli including heat, *N*-acyl amides, arachidonic acid (AA) derivatives, vanilloids, protons and cannabinoids (De Petrocellis et al., 2017).

Two agonists, capsaicin and resiniferatoxin (RTX), potently activate TRPV1 and evoke strong burning sensations. Upon activation, calcium preferentially moves through the pore, enters the cell and stimulates a series of calcium-dependent processes that ultimately lead to desensitization of the channel. Upon desensitization, the channel enters a refractory period in which it can no longer respond to further stimulation, leading to the paradoxical analgesic effect of these compounds (Iannotti et al., 2014). However, capsaicin and RTX can cause ablation of the nociceptive terminals. This, in turn, can cause a loss of the ability to identify potential tissue-damaging stimuli in the future (Chung and Campbell, 2016). Due to this, different avenues have been, and are currently, being explored to find ways to desensitize the channel without painful or ablative effects.

One avenue that has been explored is the modulation of TRP channels by cannabinoids. Endocannabinoids are the endogenous ligands that activate the CB1 and CB2 receptors, but they also activate the ionotropic cannabinoid receptors. AEA (Figure 4), an *N-*acyl amide, was the first endogenous agonist identified to activate TRPV1 (Zygmunt et al., 1999). AEA has a similar binding affinity as capsaicin, although capsaicin is significantly more potent (Storozhuk and Zholos, 2018). Palmitoylethanolamide (PEA, Figure 4), a congener of AEA, has low affinity for both CB1 and CB2 receptors, but activates TRPV1, albeit at very high concentrations (Petrosino et al., 2016). However, Petrosino and colleagues have shown that PEA enhances the effects of AEA at both the cannabinoid receptors and TRPV1 by inhibiting the degradation of AEA (De Petrocellis and Di Marzo, 2005; Petrosino et al., 2016).

Other *N*-acyl amides have also shown activity at TRPV1. AEA analogs, such as NADA and *N-*oleoyl DA (OLDA), are structurally similar to both capsaicin and AEA and have been shown to activate TRPV1 and TRPV4 receptors (Huang et al., 2002; Raboune et al., 2014). *N-*acyl GABA (NGABA), *N-*acyl aspartic acid (NAsp), *N-*acyl glycine (NGly), and *N-*acyl serine (NSer) also have significant agonist activity at TRPV1 (Raboune et al., 2014).

In addition to capsaicin and RTX, many other stimuli including heat, protons, and phytocannabinoids can activate TRPV1 (Millan, 1999; Vandewauw et al., 2018). As reported by Bisogno and colleagues in 2001, CBD was shown to act as an agonist of TRPV1 in HEK—TRPV1 cells without the ablative effects of capsaicin and RTX (Bisogno et al., 2001; De Petrocellis et al., 2011b). Another study performed by Ligresti et al. (2006) suggests that CBD can induce apoptosis in breast carcinoma cells through either direct or indirect activation of CB_2_ and/or TRPV1. CBD and its phytocannabinoid analog cannabidivarin (CBDV, Figure 5) have been shown to act as negative allosteric modulators of CB1 (Laprairie et al., 2015) or in a CB1-independent manner, respectively (Hill et al., 2013). CBD has been reported to activate TRPV1 at low micromolar concentrations similar to CBDV, and although CBDV is a weaker TRPV1 agonist than capsaicin, it still retains a high potency at TRPV1 (Iannotti et al., 2014). In line with these findings, CBD has been proven to exert anti-hyperalgesic benefits that may result from underlying activation and desensitization of TRPV1 at the peripheral and spinal level. This suggests that CBD may have therapeutic potential against inflammatory and chronic pain (De Petrocellis et al., 2011b). While many other phytocannabinoids show very weak and often barely measurable efficacies, CBD and cannabigerol (CBG, Figure 5) have been shown to be the most potent at TRPV1 and TRPM8 (De Petrocellis et al., 2011b). Furthermore, in a study of the effects of cannabinoids and cannabinoid-enriched *Cannabis* extracts on ionotropic TRP channels, De Petrocellis et al. (2011b) found that Δ^9^-tetrahydrocannabivarin (THCV, Figure 5) and cannabigevarin (CBGV, Figure 5) also stimulated TRPV1, while their acid analogs (CBDA, CBGA) stimulated TRPV1 to a lesser extent. Δ^9^-THC and its acid metabolite, Δ^9^-THCA, were not found to modulate the channel. Likewise, cannabichromene (CBC, Figure 5) and cannabinol (CBN, Figure 5) were shown to have very low efficacies at TRPV1 (De Petrocellis et al., 2011b).

Synthetic cannabinoids can also modulate TRPV1. For instance, in a collaborative effort between academia and the pharmaceutical industry, Soethoudt et al. (2017) studied the pharmacology of diverse CB2 ligands. In this work, 11 synthetic cannabinoids were tested on the ionotropic cannabinoid receptors. Putative factors in synthetic cannabinoid ligand binding at TRPV1 seem to be different among classes of synthetic cannabinoids. While binding modes for these ligands remain unknown currently some structural features can be elucidated from the scarce SAR reported. The phytocannabinoid synthetic analogs HU308, HU910, and JWH133 (Figure 6), which activate CB2 receptors, were found to weakly modulate TRPV1. Aminoalkylindole and arylpyrazole derivatives, well-known cannabinoid synthetic scaffolds, were also evaluated at these channels (Soethoudt et al., 2017). Among the aminoalkylindoles tested, the CB1/CB2 ligand WIN55,212-2 (Figure 6), was found to be the most efficacious TRPV1 ligand (Soethoudt et al., 2017). Both enantiomers of the aminoalkylindole AM1241 (*S* and *R*, Figure 6) had low efficacies at TRPV1, whereas AM630 did not appear to modulate TRPV1 at measurable values. This may indicate that the dihydro-oxazine indole core of WIN55,212-2 might be crucial for optimized TRPV1 activity, while bulky aminoalkylindole substituents and electron withdrawing phenyl substituents may also play a role in this channel. Moreover, arylpyrazoles SR141716A, SR144528, AM251, and Gp-1a (Figure 6) were also assessed at these channels. SR141716A was found to be a partial agonist of TRPV1, while Gp-1a was able to desensitize TRPV1 in the low micromolar range (De Petrocellis et al., 2001; Soethoudt et al., 2017). SR144528 and AM251 failed to modulate this channel. These results prompt us to speculate that the role of the chlorine in the chlorophenyl moiety of SR141716A, which is an iodine in AM251, is essential, the latter halogen being too bulky. Moreover, the rigidity conferred to the molecule by the tricycle in Gp-1a also decreases activity, while the bulkier pyrazole substituents of SR144528 totally abolished activity at this channel. Further studies need to be done to see how these structural changes affect the binding mode within the TRPV1 pocket.

Table 1 below summarizes all these data on endo-, phyto-, and synthetic cannabinoids that have been tested at TRPV1, along with their potencies, efficacies, and desensitization values.

**Table 1:** Functionality of phytogenic, endogenous, and synthetic cannabinoid ligands at transient receptor potential vanilloid 1 (TRPV1).

	TRPV1						
Compound	Functionality	Efficacy* (μM)	Potency EC_50_ (μM)	Desensitization** (μM)	Cell type	References
2-AG	Agonist	59.1 ± 0.3	0.85 ± 0.06	0.75 ± 0.03	TRPV1-HEK-293	Lowin and Straub (2015) and Petrosino et al. (2016)
AEA	Agonist	53.8 ± 0.2	0.27 ± 0.01	0.21 ± 0.06	TRPV1-HEK-293	Lowin and Straub (2015) and Petrosino et al. (2016)
NADA	Agonist	73.1 ± 6.4	0.040 ± 0.006	1.5 ± 0.3	TRPV1-HEK-293	Huang et al. (2002)
OLDA	Agonist	62.1 ± 5.5	36.0 ± 0.9	-	TRPV1-HEK-293	Chu et al. (2003)
PEA	Agonist (entourage effect)	-	>10	-	TRPV1-CHO	Ambrosino et al. (2013)
NGABA	Agonist	153.08 ± 10.25	-	-	TRPV1-HEK-293	Raboune et al. (2014)
NGly	Agonist	85.61 ± 16.80	-	-	TRPV1-HEK-293	Raboune et al. (2014)
NAsp	Agonist	95.13 ± 10.24	-	-	TRPV1-HEK-293	Raboune et al. (2014)
NSer	Agonist	128.76 ± 29.90	-	-	TRPV1-HEK-293	Raboune et al. (2014)
CBD	Agonist	44.7 ± 0.02	1.0 ± 0.1	0.6 ± 0.05	TRPV1-HEK-293	De Petrocellis et al. (2011b)
CBDA	Agonist	<10	19.7 ± 3.9	89.1 ± 0.3	TRPV1-HEK-293	De Petrocellis et al. (2011b)
CBDV	Agonist	21.4 ± 0.6	3.6 ± 0.7	10.0 ± 0.5	TRPV1-HEK-293	De Petrocellis et al. (2011b)
CBG	Agonist	33.8 ± 2.3	1.3 ± 0.5	2.6 ± 0.2	TRPV1-HEK-293	De Petrocellis et al. (2011b)
CBGA	Agonist	72.8 ± 2.0	21.0 ± 1.25	20.6 ± 0.5	TRPV1-HEK-293	De Petrocellis et al. (2011b)
CBGV	Agonist	58.8 ± 0.9	2.0 ± 0.1	2.3 ± 0.3	TRPV1-HEK-293	De Petrocellis et al. (2011b)
THC	-	<10	ND	ND	TRPV1-HEK-293	De Petrocellis et al. (2011b)
THCA	-	<10	ND	19.2 ± 5.3	TRPV1-HEK-293	De Petrocellis et al. (2011b)
THCV	Agonist	68.0 ± 1.6	1.5 ± 0.2	1.3 ± 0.1	TRPV1-HEK-293	De Petrocellis et al. (2011b)
THCVA	Agonist	20.0 ± 0.5	25.6 ± 1.1	>50	TRPV1-HEK-293	De Petrocellis et al. (2011b)
CBC	-	<10	24.2 ± 3.1	>50	TRPV1-HEK-293	De Petrocellis et al. (2011b)
CBN	-	<10	6.2 ± 3.7	81.7 ± 9.0	TRPV1-HEK-293	De Petrocellis et al. (2011b)
SR141716A	Agonist	17.5 ± 1.5	12.0 ± 0.6	33.7 ± 6.6	TRPV1-HEK-293	Soethoudt et al. (2017)
SR144528	-	<10	NA	>100	TRPV1-HEK-293	Soethoudt et al. (2017)
AM251	-	<10	NA	>50	TRPV1-HEK-293	Soethoudt et al. (2017)
Gp-1a	Agonist	<10	NA	3.0 ± 0.1	TRPV1-HEK-293	Soethoudt et al. (2017)
WIN55,212-2	Agonist	44.4 ± 0.9	19.2 ± 1.3	35.8 ± 2.2	TRPV1-HEK-293	Soethoudt et al. (2017)
(*R*)-AM1241	Agonist	10.7 ± 1.5	>50	>50	TRPV1-HEK-293	Soethoudt et al. (2017)
(*S*)-AM1241	Agonist	12.0 ± 1.2	>50	>50	TRPV1-HEK-293	Soethoudt et al. (2017)
AM630	-	<10	NA	>100	TRPV1-HEK-293	Soethoudt et al. (2017)
HU308	Agonist	<10	NA	69.0 ± 5.7	TRPV1-HEK-293	Soethoudt et al. (2017)
HU910	-	<10	NA	>100	TRPV1-HEK-293	Soethoudt et al. (2017)
JWH133	Agonist	24.6 ± 0.4	8.2 ± 0.7	77.7 ± 3.0	TRPV1-HEK-293	Soethoudt et al. (2017)

*Efficacy as % of ionomycin 4 μM. **Desensitization vs. standardized agonist (0.1 μM capsaicin) at IC_50_ concentrations. NA, No activity; ND, Not determined.

## TRPV2

The second member of the vanilloid subfamily, TRPV2, shares 50% sequence identity with TRPV1. TRPV2 is widely expressed in a subpopulation of medium and large diameter sensory neurons (Vay et al., 2012; Caterina, 2014). TRPV2 is insensitive to protons and capsaicin, but can be activated by high temperatures and inflammation (De Petrocellis et al., 2017). Similar to TRPV1, the activation and desensitization of TRPV2 is deeply involved in inflammatory and chronic pain (Levine and Alessandri-Haber, 2007). Therefore, finding cannabinoid ligands that can activate and subsequently desensitize TRPV2 may be a desirable therapeutic strategy.

While TRPV1 is activated by endogenous, phytogenic, and synthetic cannabinoids, TRPV2 is mainly activated by phytocannabinoids (De Petrocellis et al., 2017; Soethoudt et al., 2017). Two *N-*acyl amides, *N-*acyl proline (NPro) and *N-*acyl tyrosine (NTyr), are the only *N-*acyl amides that have been shown to activate TRPV2 with any significance (Raboune et al., 2014). Endogenous ligands such as AEA, 2-arachidonoylglycerol (2-AG, Figure 4), and NADA barely elicit a response from TRPV2 (Qin et al., 2008).

CBD has been found to be the most potent and efficacious phytocannabinoid that activates TRPV2, although at slightly lower values than at TRPV1 (Qin et al., 2008; De Petrocellis et al., 2011b). CBC (Figure 5) and the carboxylic acid derivatives CBGA and CBDA (Figure 5) are inactive at TRPV2, while the acid metabolite of Δ^9^-THC, Δ^9^ -THCA (Figure 5), has a weaker potency (De Petrocellis et al., 2011b). Δ^9^-THC has been identified as the most potent phytocannabinoid at TRPV2, although it is not selective, as it also activates TRPA1 (Qin et al., 2008). Conversely, an analog of Δ^9^-THC, 11-OH-Δ^9^-THC was found to have a low response at TRPV2, suggesting the hydroxy group somehow disrupts the activation and/or binding mode of Δ^9^-THC. However, THCV, containing a shortened alkyl side chain (propyl vs. pentyl), and Δ^9^-THCA, both act as agonists at TRPV2 with the best desensitizing ligand being THCV (De Petrocellis et al., 2011b). This suggests that the THC scaffold is robust enough to withstand moderate changes and still maintain activity at TRPV2. Further structure-activity relationships on this chemotype may allow fine-tuning of phytocannabinoid activity at this channel.

Wanting to expand on the identity of cannabinoids that activate TRPV2, Qin et al. (2008) tested a variety of synthetic cannabinoids including the THC mimics, nabilone and CP55940 (Figure 6, Table 2). Both had comparable response rates at 58% and 42%, respectively, and were the most responsive of the synthetic cannabinoids tested. The synthetic phytocannabinoid analog JWH133 (Figure 6), a potent and selective CB2 agonist, was also determined to have a very low response rate at rat TRPV2 (Qin et al., 2008). The more commonly used aminoalkylindole derivative, WIN55,212-2, was shown to have no, or very weak, response in rat TRPV2, but maintained a relatively high response rate in rat TRPA1 (Qin et al., 2008).

**Table 2:** Functionality of phytogenic, endogenous, and synthetic cannabinoid ligands at TRPV2.

TRPV2						
Compound	Functionality	Efficacy* (μM)	Potency EC_50_ (μM)	Desensitization** (μM)	Cell type	References
AEA	Agonist	NA	-	-	TRPV2-HEK-293	Qin et al. (2008)
2-AG	Agonist	29	-	-	TRPV2-HEK-293	Qin et al. (2008)
NPro	Agonist	73.35 ± 2.20	-	-	TRPV2-HEK-293	Raboune et al. (2014)
NTyr	Agonist	74.78 ± 15.21	-	-	TRPV2-HEK-293	Raboune et al. (2014)
CBD	Agonist	40.5 ± 1.6	1.25 ± 0.23	4.5 ± 0.7	TRPV2-HEK-293	De Petrocellis et al. (2011b)
CBDA	-	<10	ND	114.0 ± 18.0	TRPV2-HEK-293	De Petrocellis et al. (2011b)
CBDV	Agonist	49.9 ± 0.9	7.3 ± 0.4	31.1 ± 0.2	TRPV2-HEK-293	De Petrocellis et al. (2011b)
CBG	Agonist	73.6 ± 1.2	1.72 ± 0.08	1.5 ± 0.2	TRPV2-HEK-293	De Petrocellis et al. (2011b)
CBGA	-	<10	ND	87.3 ± 1.2	TRPV2-HEK-293	De Petrocellis et al. (2011b)
CBGV	Agonist	75.4 ± 2.4	1.41 ± 0.36	0.7 ± 0.06	TRPV2-HEK-293	De Petrocellis et al. (2011b)
CBC	-	<10	ND	6.5 ± 1.6	TRPV2-HEK-293	De Petrocellis et al. (2011b)
CBN	Agonist	39.9 ± 2.1	19.0 ± 3.7	15.7 ± 2.1	TRPV2-HEK-293	De Petrocellis et al. (2011b)
THC	Agonist	53.0 ± 1.4	0.65 ± 0.05	0.8 ± 0.1	TRPV2-HEK-293	De Petrocellis et al. (2011b)
		98	15.5	-	TRPV2-HEK-293	Qin et al. (2008)
11-OH-THC	-	57	-	-	TRPV2-HEK-293	Qin et al. (2008)
THCV	Agonist	73.8 ± 1.0	4.11 ± 0.11	0.8 ± 0.5	TRPV2-HEK-293	De Petrocellis et al. (2011b)
THCA	Agonist	68.2 ± 1.0	18.4 ± 0.9	9.8 ± 2.6	TRPV2-HEK-293	De Petrocellis et al. (2011b)
SR141716A	-	<10	NA	>100	TRPV2-HEK-293	Soethoudt et al. (2017)
SR144528	-	<10	NA		TRPV2-HEK-293	Soethoudt et al. (2017)
AM251	-	<10	NA	18.4 ± 3.5	TRPV2-HEK-293	Soethoudt et al. (2017)
Gp-1a	-	<10	NA	11.9 ± 0.7	TRPV2-HEK-293	Soethoudt et al. (2017)
WIN55,212-2	-	NA	-	-	TRPV2-HEK-293	Qin et al. (2008)
(*R*)-AM1241	Agonist	14.5 ± 0.3	12.0 ± 3.1	35.5 ± 1.5	TRPV2-HEK-293	Soethoudt et al. (2017)
(*S*)-AM1241	Agonist	11.6 ± 0.1	5.0 ± 0.1	20.6 ± 3.1	TRPV2-HEK-293	Soethoudt et al. (2017)
AM630	-	<10	NA	35.6 ± 1.4	TRPV2-HEK-293	Soethoudt et al. (2017)
HU308	-	<10	NA	>100	TRPV2-HEK-293	Soethoudt et al. (2017)
HU910	-	<10	NA	>100	TRPV2-HEK-293	Soethoudt et al. (2017)
JWH133	-	4	-	-	TRPV2-HEK-293	Qin et al. (2008)
CP55940	Agonist	42	-	-	TRPV2-HEK-293	Qin et al. (2008)
Nabilone	Agonist	58	-	-	TRPV2-HEK-293	Qin et al. (2008)

*Efficacy as % of ionomycin 4 μM. **Desensitization vs. standardized agonist (3 μM lysophosphatidylcholine) at IC_50_ concentrations. NA, No activity; ND, Not determined.

In summary, the data reported thus far (see Table 2) indicates that Δ^9^-THC, its mimics, and derivatives, have the best efficacies at TRPV2, with the exception of 11-OH-Δ^9^-THC. These results could be further expanded upon and utilized to develop new and highly selective TRPV2 agonists.

## TRPV3

The third member of the vanilloid subfamily, TRPV3, shares a 43% sequence homology with TRPV1 and is predominantly expressed in the DRG, trigeminal ganglia, and in the brain, as well as, several peripheral tissues such as testis, skin and tongue (De Petrocellis et al., 2017). The role of this channel is directly related to the perception of pain and itch. TRPV3 also acts as a thermosensor of innocuous warm temperatures (33–39°C; Pedersen et al., 2005). In addition to being activated by innocuous warm temperatures, the cooling-agent camphor and carvacrol, found in the oil of oregano and thyme, can also activate this channel (Caterina, 2014).

In contrast to TRPV1, only a few studies have demonstrated the activity of cannabinoids in this thermosensitive channel. So far, no canonical endocannabinoid has been reported to target TRPV3. However, in a recent study using endogenous lipids structurally related to AEA, *N*-acyl valine (NVal) mixtures were shown to exhibit antagonistic activity at this channel (Raboune et al., 2014). In particular, *N*-docosahexaenoyl, *N*-linoleoyl, *N*-oleoyl, and *N*-stearoyl valine were identified as individual hit antagonists, whereas no agonist was discovered among the lipids tested (Raboune et al., 2014).

When De Petrocellis et al. (2012a) tested 12 phytocannabinoids against TRPV3, they found that 10 of them exerted significant elevation of intracellular calcium, but that CBD and THCV were able to modulate TRPV3 with an efficacy similar to that of its typical agonist, carvacrol (Table 3). The authors reported that while these two phytocannabinoids potently activate TRPV3, cannabigerovarin (CBGV) and CBG acid (CBGA) were significantly more efficacious at desensitizing this channel to subsequent carvacrol activation, suggesting that the CBG scaffold may serve as a structural basis to develop TRPV3 desensitizers (De Petrocellis et al., 2012a).

**Table 3:** Functionality of phytogenic, endogenous, and synthetic cannabinoid ligands at TRPV3.

	TRPV3					
Compound	Functionality	Efficacy* (μM)	Potency EC_50_ (μM)	Desensitization** (μM)	Cell type	References
NVal	Antagonist	-	-	39.73 ± 4.16	TRPV3-HEK-293	Raboune et al. (2014)
CBD	Agonist	50.1 ± 4.8	3.7 ± 1.6	0.9 ± 0.3	TRPV3-HEK-293	De Petrocellis et al. (2012a)
THCV	Agonist	72.4 ± 2.4	3.8 ± 0.4	3.0 ± 0.2	TRPV3-HEK-293	De Petrocellis et al. (2012a)
CBGA	Agonist	17.5 ± 1.3	12.6 ± 0.2	7.4 ± 1.2	TRPV3-HEK-293	De Petrocellis et al. (2012a)
CBGV	Agonist	23.5 ± 1.7	2.4 ± 0.8	0.8 ± 0.04	TRPV3-HEK-293	De Petrocellis et al. (2012a)
SR141716A	Agonist	38.9 ± 2.1	0.85 ± 0.15	3.4 ± 0.4	TRPV3-HEK-293	Soethoudt et al. (2017)
SR144528	-	<10	NA	>100	TRPV3-HEK-293	Soethoudt et al. (2017)
AM251	Agonist	25.9 ± 1.3	0.6 ± 0.1	>50	TRPV3-HEK-293	Soethoudt et al. (2017)
Gp-1a	-	<10	NA	22.6 ± 3.9	TRPV3-HEK-293	Soethoudt et al. (2017)
WIN55,212-2	Agonist	22.9 ± 0.6	6.5 ± 0.9	>100	TRPV3-HEK-293	Soethoudt et al. (2017)
(*R*)-AM1241	Agonist	12.9 ± 0.1	10.0 ± 0.1	>50	TRPV3-HEK-293	Soethoudt et al. (2017)
(*S*)-AM1241	Agonist	16.2 ± 0.1	9.0 ± 0.1	>50	TRPV3-HEK-293	Soethoudt et al. (2017)
AM630	-	<10	NA	>100	TRPV3-HEK-293	Soethoudt et al. (2017)
HU308	-	<10	NA	>100	TRPV3-HEK-293	Soethoudt et al. (2017)
HU910	Agonist	31.3 ± 2.2	0.12 ± 0.05	12.9 ± 4.2	TRPV3-HEK-293	Soethoudt et al. (2017)
JWH133	-	<10	NA	80.6 ± 1.4	TRPV3-HEK-293	Soethoudt et al. (2017)

*Efficacy as % of ionomycin 4 μM. **Desensitization vs. standardized agonist (carvacrol) at EC_50_ concentrations or IC_50_ for antagonism. NA, No activity.

Synthetic cannabinoids have only recently been tested at TRPV3 (Soethoudt et al., 2017). The 1,1-dimethylheptyl phytocannabinoid derivative HU-910 was shown to activate TRPV3 with submicromolar potency. Interestingly, this compound does not modulate any other TRP channel tested. Other compounds such as the arylpyrazoles SR141716A and AM251 showed agonist activity at TRPV3, however, they are not selective since they also exhibited activity at other TRP channels.

Since studies of TRPV3 and its interactions with cannabinoids are limited, further investigation is required to aid in the elucidation of key structural features within each cannabinoid ligand subclass. These discoveries could be used to develop new synthetic cannabinoids that lead to more potent compounds that act at this channel. However, unlike TRPV1, TRPV3 has been shown to exhibit sensitization in response to repetitive heat stimuli (Chung et al., 2004). Due to this, studies should be performed to determine if ligand activation of TRPV3 causes a similar sensitization effect as heat activation, in which case, antagonists would be better suited for this channel.

## TRPV4

The fourth and final member of the vanilloid subfamily discussed here shares over 40% sequence homology with TRPV1 (Nilius et al., 2004). This receptor is widely expressed throughout the body and can be found in the central nervous system, epithelial cells, osteoblasts, blood vessels, and many other tissues including those of the heart, liver, and kidney (Nilius and Owsianik, 2011). TRPV4 is involved in the regulation of systemic osmotic pressure in the brain, and plays a role in vascular function, skin barrier function and nociception (Strotmann et al., 2000; Liedtke, 2005; Nilius and Owsianik, 2011). Similar to TRPV3, this channel responds to warm thermal changes, being activated by temperatures from 25°C to 34°C. In addition to diverse exogenous and endogenous ligands, TRPV4 is also activated by mechanical and osmotic stimuli (Vincent and Duncton, 2011; Duncton, 2015).

In 2003, Watanabe and coworkers reported the first experiments that linked endogenous cannabinoids to TRPV4 modulation. The authors proposed that the most abundant endocannabinoids, AEA and 2-AG, are able to activate this channel. This robust activation of TRPV4 is suggested to be due to AA metabolites formed by cytochrome P450, such as epoxyeicosatrienoic acids (Watanabe et al., 2003). Though further research is needed to unravel structural determinants of ligand-receptor interactions, the epoxy group generated upon epoxygenase metabolism of the polyunsaturated fatty acids found in endocannabinoids may be essential for ligand activity. Moreover, in the previously mentioned study of endogenous lipids, certain *N*-acyl amides were identified as TRPV4 modulators (Raboune et al., 2014). Among them, NTyr and *N*-acyl tryptophan (NTrp) mixtures stand out because of their agonist activity at TRPV4.

Concerning plant-derived cannabinoids, De Petrocellis et al. (2012a) discovered that specific compounds are also able to evoke intracellular Ca^2+^ response in cells expressing TRPV4. As depicted in Table 4, phytogenic analogs of CBD and Δ^9^-THC bearing a propyl side chain, CBDV and THCV, showed the highest efficacy and potency among the phytocannabinoids tested. These results may prompt consideration of the structural importance of cannabinoid lipophilic side chains and their interactions at TRPV4.

**Table 4:** Functionality of phytogenic, endogenous, and synthetic cannabinoid ligands at TRPV4.

	TRPV4						
Compound	Functionality	Efficacy* (μM)	Potency EC_50_ (μM)	Desensitization** (μM)	Cell type	References
AEA	Agonist (Indirect activation)	-	-	-	TRPV4-HEK-293	Watanabe et al. (2003)
2-AG	Agonist (Indirect activation)	-	-	-	TRPV4-HEK-293	Watanabe et al. (2003)
NTyr	Agonist	-	55.59 ± 7.79	-	TRPV4-HEK-293	Raboune et al. (2014)
NTrp	Agonist	-	75.59 ± 7.79	-	TRPV4-HEK-293	Raboune et al. (2014)
CBDV	Agonist	30.2 ± 0.9	0.9 ± 0.1	2.9 ± 0.3	TRPV4-HEK-293	De Petrocellis et al. (2012a)
THCV	Agonist	59.8 ± 1.7	6.4 ± 0.7	3.2 ± 0.2	TRPV4-HEK-293	De Petrocellis et al. (2012a)
CBG	Agonist	23.7 ± 1.8	5.1 ± 1.6	1.3 ± 0.1	TRPV4-HEK-293	De Petrocellis et al. (2012a)
CBGA	Agonist	36.5 ± 1.9	28.8 ± 0.3	3.6 ± 0.3	TRPV4-HEK-293	De Petrocellis et al. (2012a)
CBGV	Agonist	26.1 ± 1.7	22.2 ± 3.7	1.8 ± 0.1	TRPV4-HEK-293	De Petrocellis et al. (2012a)
CBN	Agonist	15.3 ± 1.5	16.1 ± 4.5	5.4 ± 0.8	TRPV4-HEK-293	De Petrocellis et al. (2012a)
SR141716A	-	<10	NA	2.0 ± 0.1	TRPV4-HEK-293	Soethoudt et al. (2017)
SR144528	-	<10	NA	>100	TRPV4-HEK-293	Soethoudt et al. (2017)
AM251	-	<10	NA	1.2 ± 0.1	TRPV4-HEK-293	Soethoudt et al. (2017)
Gp-1a	-	<10	NA	2.2 ± 0.1	TRPV4-HEK-293	Soethoudt et al. (2017)
WIN55,212-2	-	<10	NA	16.1 ± 1.7	TRPV4-HEK-293	Soethoudt et al. (2017)
(*R*)-AM1241	-	<10	NA	8.7 ± 0.5	TRPV4-HEK-293	Soethoudt et al. (2017)
(*S*)-AM1241	-	<10	NA	8.6 ± 0.3	TRPV4-HEK-293	Soethoudt et al. (2017)
AM630	-	<10	NA	3.2 ± 0.1	TRPV4-HEK-293	Soethoudt et al. (2017)
HU308	-	<10	NA	>100	TRPV4-HEK-293	Soethoudt et al. (2017)
HU910	-	<10	NA	>100	TRPV4-HEK-293	Soethoudt et al. (2017)
JWH133	-	13.6 ± 0.8	12.0 ± 3.0	>100	TRPV4-HEK-293	Soethoudt et al. (2017)

*Efficacy as % of ionomycin 4 μM. **Desensitization vs. standardized agonist (4-α-phorbol-12,13-didecanoate, 4αPDD) at EC_50_ concentrations. NA, No activity.

On the other hand, phytocannabinoids such as CBG, CBGA, CBGV, and CBN (Figure 5) were more readily able to desensitize this channel (after activation by 4-α-phorbol-12,13-didecanoate, 4α-PDD), even though these phytocannabinoids exhibited low efficacy and/or potency as activators of this channel. It is interesting to highlight that CBC reduced TRPV4 expression in the jejunum and ileum of mice treated with a gastrointestinal inflammatory agent, but not in control mice (De Petrocellis et al., 2012a).

Synthetic cannabinoid derivatives from representative structural families, such as aminoalkyindoles or arylpyrazoles have also been tested at this channel (Soethoudt et al., 2017). These ligands, including the CB_1_/CB_2_ agonist WIN55212-2, the cannabinoid inverse agonists SR141716A and SR144528, and the CB_2_ selective agonists HU-308 and HU-910 all failed to stimulate TRPV4 in the reported assays (Table 4).

More cannabinoids remain to be tested at this channel to determine the relevance of TRPV4 within the cannabinoid system.

## TRPA1

The first and only member of the ankyrin family to be discussed in this review is TRPA1. Members of this family are named for their extensive ARDs. TRPA1 itself contains 16 ankyrin repeat units in comparison to the six that TRPV1 contains (Paulsen et al., 2015). TRPA1 can be found co-expressed with TRPV1 in a subset of peripheral sensory neurons and is activated by pungent compounds found in mustard, garlic, and onion. These pungent compounds, called isothiocyanates, are electrophiles that covalently bind to cysteine or lysine residues found in the ARD (Caterina, 2014; Paulsen et al., 2015). TRPA1 channels have also been shown to mediate mechanical and bradykinin-evoked hyperalgesia, playing an important role in neuropathic and inflammatory pain (Yekkirala, 2013). In addition to these various ligands, TRPA1 is also activated by temperatures below 17°C, putting it at the low end of the thermo-TRP scale (Vay et al., 2012).

Very few endocannabinoids have shown activity at TRPA1. AEA was determined to have a very high efficacy (~159%) when compared to the typical TRPA1 agonist, mustard oil isothiocyanates (MO), and AEA and AA were both found to exhibit low micromolar potencies (De Petrocellis et al., 2012b; Redmond et al., 2014). Currently, these are the only two endocannabinoids with reported activity at this channel, which leaves room to discover other endogenous ligands.

In contrast to the few endocannabinoids that act at TRPA1, many phyto- and synthetic cannabinoids have been reported to activate this channel. De Petrocellis et al. (2008) tested various phytocannabinoids in TRPA1-HEK-293 cells and found that CBC, CBD, Δ^9^-THCA, CBDA, and CBG all increased intracellular Ca^2+^ levels. When the efficacy of CBC, Δ^9^-THC, and CBG was tested, it was shown that these three phytocannabinoids are more efficacious than MO. However, Δ^9^-THCA and CBDA are considered to be partial agonists of TRPA1, since they were determined to have a slightly lower efficacy than MO (De Petrocellis et al., 2008). The most potent of the phytocannabinoids initially tested were CBC, CBD, and CBN with EC_50_ values of 90 nM, 110 nM, and 180 nM respectively (De Petrocellis et al., 2011b). Later, De Petrocellis et al. (2011b) tested a wider variety of phytocannabinoids and, in agreement with their previous data, found that CBC and CBD exhibited the highest potency. However, the acid derivatives, CBGA, CBDA, and Δ^9^-THCA all showed weaker activation at TRPA1 in response to subsequent application of MO, confirming their role as partial agonists. This data shows that while the acid derivatives of phytocannabinoids can still agonize the channel, it is to a lesser extent than their decarboxylated analogs.

In addition to the phytocannabinoids that have been tested, many synthetic cannabinoids have been evaluated showing activity at TRPA1. The synthetic endocannabinoid and CB_1_ agonist, arachidonyl-2’-chloroethylamine (ACEA), was shown to have a potency similar to that of AEA at TRPA1 (Akopian et al., 2008; Ruparel et al., 2011), while the arylpyrazoles SR141716A, Gp-1a, and AM251, and the aminoalkyindoles WIN55,212-2 and AM630 were determined to activate this channel more potently than ACEA (Soethoudt et al., 2017). Furthermore, HU308, HU910, (*R*)-AM1241, and SR144528 all displayed low or no desensitization ability and slightly lower potencies than the previously mentioned synthetic cannabinoids. However, the phytocannabinoid analog JWH133 was found to be one of the most efficacious synthetic cannabinoids tested at this channel with an efficacy of ~76%. These data suggest that a wide-spanning variety of synthetic cannabinoids can activate TRPA1 with low micromolar potencies. Table 5 summarizes functional data for synthetic cannabinoids tested.

**Table 5:** Functionality of phytogenic, endogenous, and synthetic cannabinoid ligands at TRPA1.

	TRPA1						
Compound	Functionality	Efficacy* (μM)	Potency EC_50_ (μM)	Desensitization** (μM)	Cell type	References
AEA	Agonist	158.7 ± 11.1	10.1 ± 1.9	21.0 ± 1.6	TRPA1-HEK-293	De Petrocellis et al. (2012b)
AA	Agonist	-	13 ± 4	-	TRPA1-HEK-293	Redmond et al. (2014)
ACEA	Agonist	-	12 ± 2.0		TRPA1-CHO	Akopian et al. (2008)
THC	Agonist	117 ± 12	0.23 ± 0.03	-	TRPA1-HEK-293	De Petrocellis et al. (2008)
THCA	Agonist	41.6 ± 2.1	2.7 ± 0.9	95.25 ± 0.01	TRPA1-HEK-293	De Petrocellis et al. (2011b)
THCV	Agonist	234.0 ± 16.5	1.5 ± 0.6	3.07 ± 0.24	TRPA1-HEK-293	De Petrocellis et al. (2011b)
THCVA	Agonist	170.2 ± 15.9	16.4 ± 2.4	13.14 ± 0.85	TRPA1-HEK-293	De Petrocellis et al. (2011b)
CBD	Agonist	115.9 ± 4.6	0.11 ± 0.05	0.16 ± 0.05	TRPA1-HEK-293	De Petrocellis et al. (2011b)
CBDA	Agonist	113.0 ± 11	5.3 ± 1.5	4.92 ± 0.09	TRPA1-HEK-293	De Petrocellis et al. (2011b)
CBDV	Agonist	105.0 ± 0.7	0.42 ± 0.01	1.29 ± 0.38	TRPA1-HEK-293	De Petrocellis et al. (2011b)
CBC	Agonist	119.4 ± 3.1	0.09 ± 0.01	0.37 ± 0.05	TRPA1-HEK-293	De Petrocellis et al. (2011b)
CBG	Agonist	99.9 ± 1.1	0.7 ± 0.03	13.0 ± 4.8	TRPA1-HEK-293	De Petrocellis et al. (2011b)
CBGA	Agonist	182.8 ± 0.2	8.4 ± 3.5	7.14 ± 0.17	TRPA1-HEK-293	De Petrocellis et al. (2011b)
CBGV	Agonist	151.4 ± 0.9	1.6 ± 0.01	2.02 ± 0.25	TRPA1-HEK-293	De Petrocellis et al. (2011b)
CBN	Agonist	83.3 ± 4.0	0.18 ± 0.02	0.40 ± 0.04	TRPA1-HEK-293	De Petrocellis et al. (2011b)
SR141716A	Agonist	67.3 ± 1.2	1.9 ± 0.1	12.5 ± 2.2	TRPA1-HEK-293	Soethoudt et al. (2017)
SR144528	Agonist	43.8 ± 1.4	8.9 ± 1.2	>100	TRPA1-HEK-293	Soethoudt et al. (2017)
AM251	Agonist	44.4 ± 0.7	0.86 ± 0.06	17.1 ± 2.2	TRPA1-HEK-293	Soethoudt et al. (2017)
Gp-1a	Agonist	83.6 ± 0.9	2.1 ± 0.1	10.4 ± 1.4	TRPA1-HEK-293	Soethoudt et al. (2017)
WIN55,212-2	Agonist	72.3 ± 0.9	2.3 ± 0.1	6.4 ± 0.6	TRPA1-HEK-293	Soethoudt et al. (2017)
(*R*)-AM1241	Agonist	19.8 ± 1.3	19.5 ± 5.8		TRPA1-HEK-293	Soethoudt et al. (2017)
(S)-AM1241	Agonist	47.5 ± 0.8	5.8 ± 0.4	40.9 ± 5.9	TRPA1-HEK-293	Soethoudt et al. (2017)
AM630	Agonist	118.0 ± 2.0	1.9 ± 0.2	3.7 ± 0.5	TRPA1-HEK-293	Soethoudt et al. (2017)
HU910	Agonist	33.1 ± 0.1	53.1 ± 1.1	>100	TRPA1-HEK-293	Soethoudt et al. (2017)
HU308	Agonist	43.1 ± 2.2	18.5 ± 3.9	>100	TRPA1-HEK-293	Soethoudt et al. (2017)
JWH133	Agonist	76.8 ± 3.8	8.5 ± 2.3	20.0 ± 3.2	TRPA1-HEK-293	Soethoudt et al. (2017)

*Efficacy as % of 100 μM allyl isothiocyanate. **Desensitization vs. standardized agonist (100 μM allyl isothiocyanate) at IC_50_ concentrations. NS, Not Significant.

TRPA1 is suggested to play a role in many different disease states and may be involved in the mediation of the therapeutic effects of cannabinoids (Romano et al., 2013; Araújo et al., 2017). Therefore, more cannabinoids should be tested at this channel in order to better elucidate structure activity relationships.

## TRPM8

The final TRP channel that will be discussed in this review resides in the melastatin subfamily: TRPM8. TRPM8 is known for its activation at temperatures below 27°C and response to “cooling” compounds such as menthol, eucalyptol, and icilin (De Petrocellis et al., 2007). Similar to TRPV1, TRPM8 is abundantly expressed in subpopulations of primary afferent neurons (De Petrocellis et al., 2007). However, in stark contrast to the other five ionotropic cannabinoid receptors at which cannabinoids typically act as agonists, TRPM8 is antagonized by cannabinoids. The juxtaposition between TRPV1 and TRPM8 is interesting in that TRPV1 undergoes activation followed by desensitization via dephosphorylation, whereas TRPM8 is regulated by being inactivated via phosphorylation through protein kinases A and C in response to cannabinoids (De Petrocellis et al., 2008).

Similar to TRPA1, there are few endocannabinoids that seem to modulate TRPM8. The endocannabinoids, AEA and NADA have been identified as the first endogenous antagonists of TRPM8 and have potencies in the submicromolar region (De Petrocellis et al., 2007). Other *N*-acyl amides have yet to be tested at TRPM8, which leaves room for more endogenous antagonists to be identified.

De Petrocellis et al. (2008), who tested numerous phytocannabinoids on all of the ionotropic cannabinoid receptors, found that of the 12 cannabinoids tested, nearly all inhibited the effects of menthol or icilin on TRPM8 with potencies in the low- to submicromolar range. CBC was the only phytocannabinoid that was found to be completely inactive at TRPM8 (De Petrocellis et al., 2008). Interestingly, CBC was shown to be the most potent cannabinoid at TRPA1 with a potency of 0.09 ± 0.01 μM (De Petrocellis et al., 2011b). Table 6 summarizes the potencies of the cannabinoids tested in comparison to either icilin or menthol.

**Table 6:** Functionality of phytogenic, endogenous, and synthetic cannabinoid ligands at TRPM8.

	TRPM8			
Compound	Functionality*	Potency IC_50_ (μM)	Cell type	References
AEA	Antagonist vs. icilin	0.15 ± 0.08	TRPM8-HEK-293	De Petrocellis et al. (2007)
	Antagonist vs. menthol	3.09 ± 0.61	TRPM8-HEK-293	De Petrocellis et al. (2007)
NADA	Antagonist vs. icilin	0.74 ± 0.35	TRPM8-HEK-293	De Petrocellis et al. (2007)
	Antagonist vs. menthol	1.98 ± 0.38	TRPM8-HEK-293	De Petrocellis et al. (2007)
THC	Antagonist vs. icilin	0.16 ± 0.01	TRPM8-HEK-293	De Petrocellis et al. (2008)
	Antagonist vs. menthol	0.15 ± 0.02	TRPM8-HEK-293	De Petrocellis et al. (2008)
THCA	Antagonist vs. icilin	0.14 ± 0.02	TRPM8-HEK-293	De Petrocellis et al. (2008)
	Antagonist vs. menthol	0.07 ± 0.01	TRPM8-HEK-293	De Petrocellis et al. (2008)
THCV	Antagonist vs. icilin	0.87 ± 0.01	TRPM8-HEK-293	De Petrocellis et al. (2011b)
THCVA	Antagonist vs. icilin	1.33 ± 0.02	TRPM8-HEK-293	De Petrocellis et al. (2011b)
CBD	Antagonist vs. icilin	0.08 ± 0.01	TRPM8-HEK-293	De Petrocellis et al. (2008)
	Antagonist vs. menthol	0.14 ± 0.01	TRPM8-HEK-293	De Petrocellis et al. (2008)
CBDA	Antagonist vs. icilin	0.9 ± 0.1	TRPM8-HEK-293	De Petrocellis et al. (2008)
	Antagonist vs. menthol	1.6 ± 0.4	TRPM8-HEK-293	De Petrocellis et al. (2008)
CBDV	Antagonist vs. icilin	0.90 ± 0.01	TRPM8-HEK-293	De Petrocellis et al. (2011b)
CBG	Antagonist vs. icilin	0.14 ± 0.01	TRPM8-HEK-293	De Petrocellis et al. (2008)
	Antagonist vs. menthol	0.16 ± 0.03	TRPM8-HEK-293	De Petrocellis et al. (2008)
CBGA	Antagonist vs. icilin	1.31 ± 0.09	TRPM8-HEK-293	De Petrocellis et al. (2011b)
CBGV	Antagonist vs. icilin	1.71 ± 0.04	TRPM8-HEK-293	De Petrocellis et al. (2011b)
CBC	Antagonist vs. icilin	40.7 ± 0.6	TRPM8-HEK-293	De Petrocellis et al. (2011b)
CBN	Antagonist vs. icilin	0.21 ± 0.05	TRPM8-HEK-293	De Petrocellis et al. (2011b)
SR141716A	Antagonist vs. icilin	0.052 ± 0.011	TRPM8-HEK-293	De Petrocellis et al. (2007)
SR144528	Antagonist vs. icilin	0.017 ± 0.005	TRPM8-HEK-293	De Petrocellis et al. (2007)
AM251	Antagonist vs. icilin	18.4 ± 3.5	TRPM8-HEK-293	Soethoudt et al. (2017)
Gp-1a	NA	>50	TRPM8-HEK-293	Soethoudt et al. (2017)
WIN55,212-2	Antagonist vs. icilin	72.9 ± 4.5	TRPM8-HEK-293	Soethoudt et al. (2017)
(*R*)-AM1241	NA	>50	TRPM8-HEK-293	Soethoudt et al. (2017)
(*S*)-AM1241	NA	>50	TRPM8-HEK-293	Soethoudt et al. (2017)
AM630	Antagonist vs. icilin	4.3 ± 0.3	TRPM8-HEK-293	Soethoudt et al. (2017)
HU308	NA	>100	TRPM8-HEK-293	Soethoudt et al. (2017)
HU910	NA	>100	TRPM8-HEK-293	Soethoudt et al. (2017)
JWH133	Antagonist vs. icilin	48.4 ± 3.5	TRPM8-HEK-293	Soethoudt et al. (2017)

*Functionality determined against 0.25 μM icilin or 50 μM menthol. NA, No activity.

Soethoudt et al. (2017) evaluated several synthetic cannabinoids at TRPM8. Aminoalkylindole derivatives, such as AM630 and AM1241, or phytocannabinoid analogs, such as HU308 or HU910, failed to modulate this channel. However, certain arylpyrazoles were able to modulate TRPM8. SR141716A and SR144528 were found to have potencies in the submicromolar range against icilin. Interestingly, SR141716A, showed activity in the nanomolar range, therefore potently modulating three of the six channels discussed in this review.

Since data on cannabinoids at TRPM8 is still sparse, further studies on its interactions with cannabinoids and the mechanism of inactivation need to be performed to fully understand the relevance of this channel.

In general terms, as we can observe from the summarized data, channel selectivity remains a challenge among cannabinoid chemotypes. Therefore, further studies should aim at the identification of novel selective TRP cannabinoids that help reveal the therapeutic potential and the mechanism of action of these ligands in the ionotropic receptors.

## Final Remarks

It has been widely demonstrated that cannabinoid ligands exert numerous physiopathological functions by modulating TRP channels. These cannabinoid-related TRP channels include members from the vanilloid, ankyrin, and melastatin subfamilies. The six channels discussed in this review are also considered thermo-TRP channels, due to their location in sensory neurons and their ability to be activated by a wide range of temperatures. The modulation of these six channels by temperature and cannabinoids is complex, and the relationship between the channels and their activation in response to cannabinoids can be further explored for various therapeutic uses, including chronic pain and inflammation. Current knowledge on how and which cannabinoids target TRP channels is still scarce, but has largely increased in the last decade. By classifying the cannabinoid structures able to modulate these receptors, we aim to provide an analysis that helps identifying key features involved in their activity at each particular channel.

Of the endocannabinoids tested at the vanilloid-type channels thus far, all act as agonists with the exception of the endogenous lipid NVal, which acts as an antagonist of TPRV4. Endogenous cannabinoids are also able to activate the ankyrin channel, TRPA1, whereas they exhibit antagonistic effects at the melastatin receptor, TRPM8. The endocannabinoid, AEA was found to be the first endogenous agonist at TRPV1 and has a submicromolar potency. AEA also acts as an agonist at TRPA1, an antagonist at TRPM8, and indirectly activates TRPV4 through its cytochrome-450 metabolites (Watanabe et al., 2003).

Several phytocannabinoids have shown remarkable results at these channels. The active compounds identified tend to activate TRPV1–4 and TRPA1, while they antagonize the activation of icilin or menthol at TRPM8. Among the phytocannabinoids tested in these six channels, CBD and THCV are the more promiscuous since they are potent and efficacious modulators of all the TRP channels discussed here. CBD and CBG are reported to be the most potent ligands tested at TRPV1. Δ^9^-THC has been found to show no channel modulation, however, Δ^9^-THC has been shown to potently activate TRPV2.

Concerning synthetic cannabinoids, so far only a few, but from representative cannabinoid scaffolds, have been tested. Arylpyrazoles such as SR141716A, SR144528, or AM251 (Figure 6) are able to activate TRPA1, while acting as TRPM8 antagonists. Even though these compounds do not show activity at the vanilloid channels TRPV2 and TRPV4, SR141716A and AM251 can weakly modulate TRPV1 and TRPV3. The aminoalkylindole chemotype has also been explored at these six channels. For instance, the widely used member of this class, WIN55,212-2, has been shown to exert some of its effects through activation of TRPV1 and TRPA1 (Ruparel et al., 2011). Moreover, phytocannabinoid synthetic derivatives such as HU308, HU910, and JWH133 have also been tested in the search of a better understanding of their pharmacological profile. While HU308 does not display potent modulation of any of the channels, other analogs in this class do. For example, JWH133 was shown to modulate TRPV1 and TRPA1 and antagonize the effects of icilin at TRPM8, while HU910 was shown to activate TRPV3. HU308 and HU910 share the dimethoxyphenyl core and the lipophilic side chain, mainly differing in the position of the aliphatic hydroxyl group. This feature may determine TRPV3 recognition. On the other hand, the tricyclic rigidity of JWH133 along with the lack of phenolic hydroxyl may define the ability of this compound to target TRPV1 and TRPA1. The structural differences highlighted here clearly effect the ability of the ligand to modulate their TRP channels, but how these changes affect the binding of the ligand in the channel has yet to be determined. A more inclusive investigation of the binding sites, as well as, the effects of changing moieties could provide insight on how to better design cannabinoid ligands for selectivity and potency.

In summary, we have shown here that a broad range of cannabinoids (endogenous, phytogenic, and synthetic cannabinoids) act at one or more of the following ionotropic channels: TRPV1, TRPV2, TRPV3, TRPV4, TRPA1 and TRPM8. This information is the first step in understanding the importance of ionotropic channels to cannabinoid effects, such as analgesia for chronic pain. However, there is much more that needs to be discovered. What residues are involved in the binding of these cannabinoids to the ionotropic cannabinoid receptors? How do these cannabinoids activate or inactivate the channels at which they act? What structural modifications will produce more potent cannabinoids at these channels? Pursuit of these research directions should lead to a better understanding of the importance of TRP channels to the physiology of the endocannabinoid system.

## Author Contributions

PR provided guidance for the creation of this manuscript and performed editing of the article. CM and PM wrote this review article and prepared all figures. All the authors substantially contributed to the redaction of the manuscript. Then, they all approved the manuscript to be published.

## Conflict of Interest Statement

The authors declare that the research was conducted in the absence of any commercial or financial relationships that could be construed as a potential conflict of interest.

## References

[B1] Ahluwalia J. Urban L. Bevan S. Nagy I. (2003). Anandamide regulates neuropeptide release from capsaicin-sensitive primary sensory neurons by activating both the cannabinoid 1 receptor and the vanilloid receptor 1 *in vitro*. Eur. J. Neurosci. 17, 2611–2618. 10.1046/j.1460-9568.2003.02703.x 12823468

[B2] Akopian A. N. Ruparel N. B. Patwardhan A. Hargreaves K. M. (2008). Cannabinoids desensitize capsaicin and mustard oil responses in sensory neurons via TRPA1 activation. J. Neurosci. 28, 1064–1075. 10.1523/JNEUROSCI.1565-06.2008 18234885 PMC6671418

[B3] Ambrosino P. Soldovieri M. V. Russo C. Taglialatela M. (2013). Activation and desensitization of TRPV1 channels in sensory neurons by the PPARα agonist palmitoylethanolamide. Br. J. Pharmacol. 168, 1430–1444. 10.1111/bph.12029 23083124 PMC3596648

[B4] Anand U. Otto W. R. Sanchez-Herrera D. Facer P. Yiangou Y. Korchev Y.. (2008). Cannabinoid receptor CB_2_ localisation and agonist-mediated inhibition of capsaicin responses in human sensory neurons. Pain 138, 667–680. 10.1016/j.pain.2008.06.007 18692962

[B5] Araújo D. S. M. Miya-Coreixas V. S. Pandolfo P. Calaza K. C. (2017). Cannabinoid receptors and TRPA1 on neuroprotection in a model of retinal ischemia. Exp. Eye Res. 154, 116–125. 10.1016/j.exer.2016.11.015 27876485

[B6] Bisogno T. Hanusae L. R. De Petrocellis L. Tchilibon S. Ponde D. E. Brandi I.. (2001). Molecular targets for cannabidiol and its synthetic analogues: effect on vanilloid VR1 receptors and on the cellular uptake and enzymatic hydrolysis of anandamide. Br. J. Pharmacol. 134, 845–852. 10.1038/sj.bjp.0704327 11606325 PMC1573017

[B7] Caterina M. J. (2014). TRP channel cannabinoid receptors in skin sensation, homeostasis, and inflammation. ACS Chem. Neurosci. 5, 1107–1116. 10.1021/cn5000919 24915599 PMC4240254

[B8] Chu C. J. Huang S. M. De Petrocellis L. Bisogno T. Ewing S. A. Miller J. D.. (2003). *N*-oleoyldopamine, a novel endogenous capsaicin-like lipid that produces hyperalgesia. J. Biol. Chem. 278, 13633–13639. 10.1074/jbc.M211231200 12569099

[B9] Chung M. K. Campbell J. N. (2016). Use of capsaicin to treat pain: mechanistic and therapeutic considerations. Pharmaceuticals 9:66. 10.3390/ph9040066 27809268 PMC5198041

[B10] Chung M. K. Lee H. Mizuno A. Suzuki M. Caterina M. J. (2004). TRPV3 and TRPV4 mediate warmth-evoked currents in primary mouse keratinocytes. J. Biol. Chem. 279, 21569–21575. 10.1074/jbc.M401872200 15004014

[B11] Cristino L. de Petrocellis L. Pryce G. Baker D. Guglielmotti V. Di Marzo V. (2006). Immunohistochemical localization of cannabinoid type 1 and vanilloid transient receptor potential vanilloid type 1 receptors in the mouse brain. Neuroscience 139, 1405–1415. 10.1016/j.neuroscience.2006.02.074 16603318

[B13] De Petrocellis L. Bisogno T. Maccarrone M. Davis J. B. Finazzi-Agrò A. Di Marzo V. (2001). The activity of anandamide at vanilloid VR1 receptors requires facilitated transport across the cell membrane and is limited by intracellular metabolism. J. Biol. Chem. 276, 12856–12863. 10.1074/jbc.m008555200 11278420

[B12] De Petrocellis L. Di Marzo V. (2005). Lipids as regulators of the activity of transient receptor potential type V1 (TRPV1) channels. Life Sci. 77, 1651–1666. 10.1016/j.lfs.2005.05.021 15936040

[B14] De Petrocellis L. Guida F. Moriello A. S. De Chiaro M. Piscitelli F. De Novellis V.. (2011a). *N*-palmitoyl-vanillamide (palvanil) is a non-pungent analogue of capsaicin with stronger desensitizing capability against the TRPV1 receptor and anti-hyperalgesic activity. Pharmacol. Res. 63, 294–299. 10.1016/j.phrs.2010.12.019 21215315

[B15] De Petrocellis L. Ligresti A. Moriello A. S. Allarà M. Bisogno T. Petrosino S.. (2011b). Effects of cannabinoids and cannabinoid-enriched Cannabis extracts on TRP channels and endocannabinoid metabolic enzymes. Br. J. Pharmacol. 163, 1479–1494. 10.1111/j.1476-5381.2010.01166.x 21175579 PMC3165957

[B16] De Petrocellis L. Nabissi M. Santoni G. Ligresti A. (2017). Actions and regulation of ionotropic cannabinoid receptors. Adv. Pharmacol. 80, 249–289. 10.1016/bs.apha.2017.04.001 28826537

[B17] De Petrocellis L. Orlando P. Moriello A. S. Aviello G. Stott C. Izzo A. A.. (2012a). Cannabinoid actions at TRPV channels: effects on TRPV3 and TRPV4 and their potential relevance to gastrointestinal inflammation. Acta Physiol. 204, 255–266. 10.1111/j.1748-1716.2011.02338.x 21726418

[B18] De Petrocellis L. Schiano Moriello A. Imperatore R. Cristino L. Starowicz K. Di Marzo V. (2012b). A re-evaluation of 9-HODE activity at TRPV1 channels in comparison with anandamide: enantioselectivity and effects at other TRP channels and in sensory neurons. Br. J. Pharmacol. 167, 1643–1651. 10.1111/j.1476-5381.2012.02122.x 22861649 PMC3525867

[B19] De Petrocellis L. Starowicz K. Moriello A. S. Vivese M. Orlando P. Di Marzo V. (2007). Regulation of transient receptor potential channels of melastatin type 8 (TRPM8): effect of cAMP, cannabinoid CB_1_ receptors and endovanilloids. Exp. Cell Res. 313, 1911–1920. 10.1016/j.yexcr.2007.01.008 17428469

[B20] De Petrocellis L. Vellani V. Schiano-Moriello A. Marini P. Magherini P. C. Orlando P.. (2008). Plant-derived cannabinoids modulate the activity of transient receptor potential channels of ankyrin type-1 and melastatin type-8. J. Pharmacol. Exp. Ther. 325, 1007–1015. 10.1124/jpet.107.134809 18354058

[B21] Duncton M. A. J. (2015). “Small molecule agonists and antagonists of TRPV4,” in TRP Channels as Therapeutic Targets, ed. Szallasi A. (San Diego, CA: Academic Press), 205–219.

[B22] Guindon J. Hohmann A. G. (2009). The endocannabinoid system and pain. CNS Neurol. Disord. Drug Targets 8, 403–421. 10.2174/187152709789824660 19839937 PMC2834283

[B23] Hellmich U. A. Gaudet R. (2014). High-resolution views of TRPV1 and their implications for the TRP channel superfamily. Handb. Exp. Pharmacol. 222, 991–1004. 10.1007/978-3-319-05161-1_11 24961977 PMC5075239

[B24] Hill T. D. M. Cascio M. G. Romano B. Duncan M. Pertwee R. G. Williams C. M.. (2013). Cannabidivarin-rich cannabis extracts are anticonvulsant in mouse and rat via a CB_1_ receptor-independent mechanism. Br. J. Pharmacol. 170, 679–692. 10.1111/bph.12321 23902406 PMC3792005

[B25] Huang S. M. Bisogno T. Trevisani M. Al-Hayan A. De Petrocellis L. Fezza F.. (2002). An endogenous capsaicin-like substance with high potency at recombinant and native vanilloid VR1 receptors. Proc. Natl. Acad. Sci. U S A 99, 8400–8405. 10.1073/pnas.122196999 12060783 PMC123079

[B26] Iannotti F. A. Hill C. L. Leo A. Alhusaini A. Soubrane C. Mazzarella E.. (2014). Nonpsychotropic plant cannabinoids, cannabidivarin (CBDV) and cannabidiol (CBD), activate and desensitize transient receptor potential vanilloid 1 (TRPV1) channels *in vitro*: potential for the treatment of neuronal hyperexcitability. ACS Chem. Neurosci. 5, 1131–1141. 10.1021/cn5000524 25029033

[B27] Laprairie R. B. Bagher A. M. Kelly M. E. M. Denovan-Wright E. M. (2015). Cannabidiol is a negative allosteric modulator of the cannabinoid CB_1_ receptor. Br. J. Pharmacol. 172, 4790–4805. 10.1111/bph.13250 26218440 PMC4621983

[B28] Levine J. D. Alessandri-Haber N. (2007). TRP channels: targets for the relief of pain. Biochim. Biophys. Acta 1772, 989–1003. 10.1016/j.bbadis.2007.01.008 17321113

[B29] Liedtke W. (2005). TRPV4 plays an evolutionary conserved role in the transduction of osmotic and mechanical stimuli in live animals. J. Physiol. 567, 53–58. 10.1113/jphysiol.2005.088963 15961428 PMC1474158

[B30] Ligresti A. Moriello A. S. Starowicz K. Matias I. Pisanti S. De Petrocellis L.. (2006). Antitumor activity of plant cannabinoids with emphasis on the effect of cannabidiol on human breast carcinoma. J. Pharmacol. Exp. Ther. 318, 1375–1387. 10.1124/jpet.106.105247 16728591

[B31] Lowin T. Straub R. H. (2015). Cannabinoid-based drugs targeting CB_1_ and TRPV1, the sympathetic nervous system, and arthritis. Arthritis Res. Ther. 17:226. 10.1186/s13075-015-0743-x 26343051 PMC4561168

[B32] Luongo L. Costa B. D’Agostino B. Guida F. Comelli F. Gatta L.. (2012). Palvanil, a non-pungent capsaicin analogue, inhibits inflammatory and neuropathic pain with little effects on bronchopulmonary function and body temperature. Pharmacol. Res. 66, 243–250. 10.1016/j.phrs.2012.05.005 22634607

[B33] Millan M. J. (1999). The induction of pain: an integrative review. Prog. Neurobiol. 57, 1–164. 10.1016/s0301-0082(98)00048-3 9987804

[B35] Morales P. Hurst D. P. Reggio P. H. (2017). Molecular targets of the phytocannabinoids: a complex picture. Prog. Chem. Org. Nat. Prod. 103, 103–131. 10.1007/978-3-319-45541-9_4 28120232 PMC5345356

[B34] Morales P. Reggio P. H. (2017). An update on non-CB_1_, non-CB_2_ cannabinoid related G-protein-coupled receptors. Cannabis Cannabinoid Res. 2, 265–273. 10.1089/can.2017.0036 29098189 PMC5665501

[B36] Morales P. Isawi I. Reggio P. H. (2018). Towards a better understanding of the cannabinoid-related orphan receptors GPR3, GPR6, and GPR12. Drug Metab. Rev. 50, 74–93. 10.1080/03602532.2018.1428616 29390908 PMC6093286

[B38] Nilius B. Owsianik G. (2011). The transient receptor potential family of ion channels. Genome Biol. 12:218. 10.1186/gb-2011-12-3-218 21401968 PMC3129667

[B39] Nilius B. Szallasi A. (2014). Transient receptor potential channels as drug targets: from the science of basic research to the art of medicine. Pharmacol. Rev. 66, 676–814. 10.1124/pr.113.008268 24951385

[B37] Nilius B. Vriens J. Prenen J. Droogmans G. Voets T. (2004). TRPV4 calcium entry channel: a paradigm for gating diversity. Am. J. Physiol. Cell Physiol. 286, C195–C205. 10.1152/ajpcell.00365.2003 14707014

[B40] Paulsen C. E. Armache J. P. Gao Y. Cheng Y. Julius D. (2015). Structure of the TRPA1 ion channel suggests regulatory mechanisms. Nature 520, 511–517. 10.1038/nature14367 25855297 PMC4409540

[B41] Pedersen S. F. Owsianik G. Nilius B. (2005). TRP channels: an overview. Cell Calcium 38, 233–252. 10.1016/j.ceca.2005.06.028 16098585

[B42] Perálvarez-Marín A. Doñate-macian P. Gaudet R. (2012). What do we know about the transient receptor potential vanilloid 2 (TRPV2) ion channel? FEBS J. 280, 5471–5487. 10.1111/febs.12302 23615321 PMC3783526

[B43] Petrosino S. Schiano Moriello A. Cerrato S. Fusco M. Puigdemont A. De Petrocellis L.. (2016). The anti-inflammatory mediator palmitoylethanolamide enhances the levels of 2-arachidonoyl-glycerol and potentiates its actions at TRPV1 cation channels. Br. J. Pharmacol. 173, 1154–1162. 10.1111/bph.13084 25598150 PMC5338153

[B44] Price T. J. Patwardhan A. Akopian A. N. Hargreaves K. M. Flores C. M. (2004). Modulation of trigeminal sensory neuron activity by the dual cannabinoid-vanilloid agonists anandamide, *N*-arachidonoyl-dopamine and arachidonyl-2-chloroethylamide. Br. J. Pharmacol. 141, 1118–1130. 10.1038/sj.bjp.0705711 15006899 PMC1574881

[B45] Qin N. Neeper M. P. Liu Y. Hutchinson T. L. Lubin M. L. Flores C. M. (2008). TRPV2 is activated by cannabidiol and mediates CGRP release in cultured rat dorsal root ganglion neurons. J. Neurosci. 28, 6231–6238. 10.1523/JNEUROSCI.0504-08.2008 18550765 PMC6670541

[B46] Raboune S. Stuart J. M. Leishman E. Takacs S. M. Rhodes B. Basnet A.. (2014). Novel endogenous *N*-acyl amides activate TRPV1–4 receptors, BV-2 microglia, and are regulated in brain in an acute model of inflammation. Front. Cell. Neurosci. 8:195. 10.3389/fncel.2014.00195 25136293 PMC4118021

[B47] Redmond W. J. Gu L. Camo M. McIntyre P. Connor M. (2014). Ligand determinants of fatty acid activation of the pronociceptive ion channel TRPA1. PeerJ 2:e248. 10.7717/peerj.248 24516781 PMC3913255

[B48] Romano B. Borrelli F. Fasolino I. Capasso R. Piscitelli F. Cascio M. G.. (2013). The cannabinoid TRPA1 agonist cannabichromene inhibits nitric oxide production in macrophages and ameliorates murine colitis. Br. J. Pharmacol. 169, 213–229. 10.1111/bph.12120 23373571 PMC3632250

[B49] Rossi F. Siniscalco D. Luongo L. De Petrocellis L. Bellini G. Petrosino S.. (2009). The endovanilloid/endocannabinoid system in human osteoclasts: possible involvement in bone formation and resorption. Bone 44, 476–484. 10.1016/j.bone.2008.10.056 19059369

[B50] Ruparel N. B. Patwardhan A. M. Akopian A. N. Hargreaves K. M. (2011). Desensitization of transient receptor potential ankyrin 1 (TRPA1) by the TRP vanilloid 1-selective cannabinoid arachidonoyl-2 chloroethanolamine. Mol. Pharmacol. 80, 117–123. 10.1124/mol.110.068940 21441412 PMC3127531

[B51] Soethoudt M. Grether U. Fingerle J. Grim T. W. Fezza F. de Petrocellis L.. (2017). Cannabinoid CB_2_ receptor ligand profiling reveals biased signalling and off-target activity. Nat. Commun. 8:13958. 10.1038/ncomms13958 28045021 PMC5216056

[B52] Storozhuk M. Zholos A. (2018). TRP channels as novel targets for endogenous ligands: focus on endocannabinoids and nociceptive signalling. Curr. Neuropharmacol. 16, 137–150. 10.2174/1570159X15666170424120802 28440188 PMC5883376

[B53] Strotmann R. Harteneck C. Nunnenmacher K. Schultz G. Plant T. D. (2000). OTRPC4, a nonselective cation channel that confers sensitivity to extracellular osmolarity. Nat. Cell Biol. 2, 695–702. 10.1038/35036318 11025659

[B54] Vandewauw I. De Clercq K. Mulier M. Held K. Pinto S. Van Ranst N.. (2018). A TRP channel trio mediates acute noxious heat sensing. Nature 555, 662–666. 10.1038/nature26137 29539642

[B55] Vay L. Gu C. McNaughton P. A. (2012). The thermo-TRP ion channel family: properties and therapeutic implications. Br. J. Pharmacol. 165, 787–801. 10.1111/j.1476-5381.2011.01601.x 21797839 PMC3312478

[B56] Vincent F. Duncton M. (2011). TRPV4 agonists and antagonists. Curr. Top. Med. Chem. 11, 2216–2226. 10.2174/156802611796904861 21671873

[B57] Watanabe H. Vriens J. Prenen J. Droogmans G. Voets T. Nillus B. (2003). Anandamide and arachidonic acid use epoxyeicosatrienoic acids to activate TRPV4 channels. Nature 424, 434–438. 10.1038/nature01807 12879072

[B58] Winter Z. Buhala A. Ötvös F. Jósvay K. Vizler C. Dombi G.. (2013). Functionally important amino acid residues in the transient receptor potential vanilloid 1 (TRPV1) ion channel—an overview of the current mutational data. Mol. Pain 9:30. 10.1186/1744-8069-9-30 23800232 PMC3707783

[B59] Woodhams S. G. Chapman V. Finn D. P. Hohmann A. G. Neugebauer V. (2017). The cannabinoid system and pain. Neuropharmacology 124, 105–120. 10.1016/j.neuropharm.2017.06.015 28625720 PMC5785108

[B60] Yekkirala A. S. (2013). Two to tango: GPCR oligomers and GPCR-TRP channel interactions in nociception. Life Sci. 92, 438–445. 10.1016/j.lfs.2012.06.021 22771696

[B61] Yin Y. Wu M. Zubcevic L. Borschel W. F. Lander G. C. Lee S. (2018). Structure of the cold- and menthol-sensing ion channel TRPM8. Science 359, 237–241. 10.1126/science.aan4325 29217583 PMC5810135

[B62] Zygmunt P. M. Petersson J. Andersson D. A. Chuang H. Sørgård M. Di Marzo V.. (1999). Vanilloid receptors on sensory nerves mediate the vasodilator action of anandamide. Nature 400, 452–457. 10.1038/22761 10440374

